# Cellular and animal models for facioscapulohumeral muscular dystrophy

**DOI:** 10.1242/dmm.046904

**Published:** 2020-10-28

**Authors:** Alec M. DeSimone, Justin Cohen, Monkol Lek, Angela Lek

**Affiliations:** Yale School of Medicine, Department of Genetics, New Haven, CT 06510, USA

**Keywords:** FSHD, DUX4, Cellular model, Animal model, Xenograft

## Abstract

Facioscapulohumeral muscular dystrophy (FSHD) is one of the most common forms of muscular dystrophy and presents with weakness of the facial, scapular and humeral muscles, which frequently progresses to the lower limbs and truncal areas, causing profound disability. Myopathy results from epigenetic de-repression of the *D4Z4* microsatellite repeat array on chromosome 4, which allows misexpression of the developmentally regulated *DUX4* gene. DUX4 is toxic when misexpressed in skeletal muscle and disrupts several cellular pathways, including myogenic differentiation and fusion, which likely underpins pathology. *DUX4* and the *D4Z4* array are strongly conserved only in primates, making FSHD modeling in non-primate animals difficult. Additionally, its cytotoxicity and unusual mosaic expression pattern further complicate the generation of *in vitro* and *in vivo* models of FSHD. However, the pressing need to develop systems to test therapeutic approaches has led to the creation of multiple engineered FSHD models. Owing to the complex genetic, epigenetic and molecular factors underlying FSHD, it is difficult to engineer a system that accurately recapitulates every aspect of the human disease. Nevertheless, the past several years have seen the development of many new disease models, each with their own associated strengths that emphasize different aspects of the disease. Here, we review the wide range of FSHD models, including several *in vitro* cellular models, and an array of transgenic and xenograft *in vivo* models, with particular attention to newly developed systems and how they are being used to deepen our understanding of FSHD pathology and to test the efficacy of drug candidates.

## Abstract (precis)

**Summary:** Owing to its complex etiology and the toxicity of DUX4, modeling facioscapulohumeral muscular dystrophy (FSHD) is uniquely challenging. Here, we review the approaches that overcame these difficulties to develop highly relevant FSHD models.

## Introduction

**Fig. 1.**
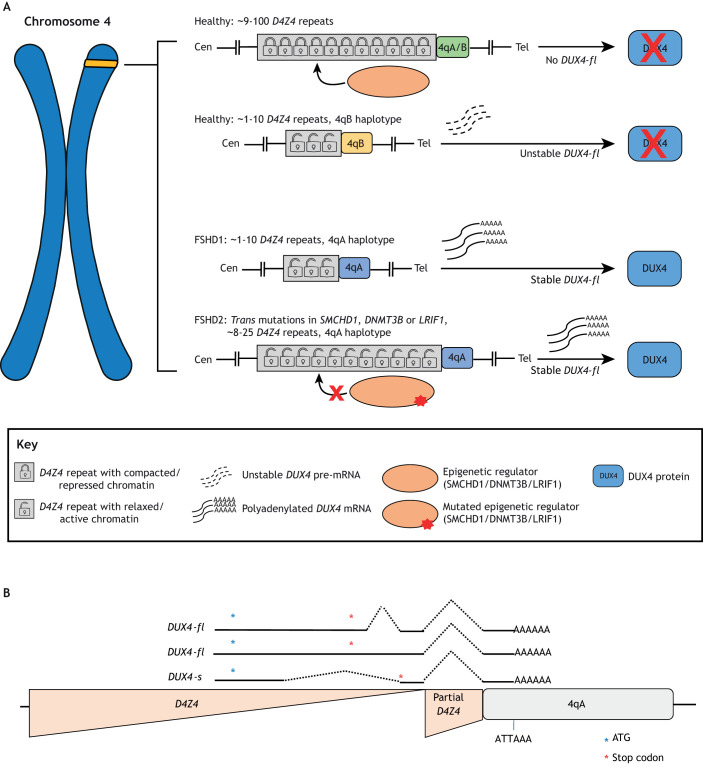
**Genetics of FSHD.** (A) The *D4Z4* array is located near the telomere of chromosome 4q. (Top panel) In most healthy individuals, the array contains ∼9-100 repeat elements and either a 4qA or 4qB haplotype, resulting in a compacted, epigenetically repressed array. (Second panel) Repeat contraction leads to array relaxation and epigenetic de-repression of the *DUX4* gene contained within each repeat. When this occurs in the presence of a 4qB haplotype, the *DUX4* transcript is unstable and disease does not occur. (Third panel) When contraction occurs in the presence of a 4qA haplotype, the *DUX4* transcript from the final repeat incorporates a polyadenylation signal. This stabilizes the RNA, allowing processing and translation of the DUX4 protein, resulting in FSHD1. (Bottom panel) *Trans*-acting mutations in the epigenetic regulators *SMCHD1*, *DNMT3B* and/or *LRIF1* can de-repress the array. In the presence of a 4qA haplotype, this allows synthesis of stable *DUX4* mRNA, resulting in FSHD2. Cen, centromere proximal side; Tel, telomere proximal side. (B) Transcription of *DUX4* from the final full *D4Z4* repeat reads into the adjacent DNA, incorporating additional material from a partial *D4Z4* repeat and the region immediately following the array. In the presence of a 4qA haplotype, this will include an ATTAAA polyadenylation site, which stabilizes the transcript. Alternate splicing of the transcript results in multiple mRNAs. Adapted from Snider et al. (2010) under the terms of the CC-BY 3.0 license.

Facioscapulohumeral muscular dystrophy (FSHD) is an autosomal dominant muscular dystrophy with asymmetric involvement that initially affects the facial muscles, then progresses to the shoulder girdle and humeral muscles, and later affects the truncal muscles and lower extremities (DeSimone et al., 2017; Padberg, 1982, PhD thesis). Progression often leads to profound disability, with more than 20% of affected individuals becoming wheelchair dependent (Statland and Tawil, 2014). It is one of the most common muscular dystrophies, estimated to affect ∼1 in 8000-20,000 individuals (Deenen et al., 2014; Mostacciuolo et al., 2009; Padberg, 1982, PhD thesis; Sposìto et al., 2005). Through the combined work of several genetic studies, the FSHD disease gene was mapped to the subtelomeric region of the short arm of chromosome 4 (4q35; reviewed in DeSimone et al., 2017). The critical region contains a repeat array consisting of tandem 3.3 kb elements known as *D4Z4* repeats (van Deutekom et al., 1993; Wijmenga et al., 1992) (Fig. 1A; see Glossary, Box 1). The number of repeats in the general population can range from approximately ten to over 100, but FSHD1, the most common form of the disease, is associated with shorter *D4Z4* arrays (Box 1). The risk of developing FSHD1 increases with decreasing numbers of repeat units, with individuals carrying seven or fewer having a high probability of disease, individuals carrying eight to ten units having moderate probability, and individuals with a larger number of units having lower probability (Lunt et al., 1995; Orrell et al., 1999; Ricci et al., 1999; Rossi et al., 2007; Sacconi et al., 2019; Schaap et al., 2013; Scionti et al., 2012; van Deutekom et al., 1993; Wijmenga et al., 1992). Allele contraction results in a host of epigenetic changes that relax the chromatin and allow expression of the genes in the region (reviewed in Greco et al., 2020; Salsi et al., 2020). Additionally, to become pathogenic, the shortened array must be present on a ‘permissive’ chromosome (Box 1) that carries a 4qA allele adjacent to the *D4Z4* array, as well as particular simple sequence length polymorphisms (Box 1) (Lemmers et al., 2002, 2004, 2007, 2010a,b; Spurlock et al., 2010; van Geel et al., 2002). In the less common form of the disease, FSHD2, array size is less important; *D4Z4* arrays in FSHD2 patients are, on average, shorter than in the unaffected population, but are most often longer than in FSHD1 (de Greef et al., 2010; Sacconi et al., 2019). Critically, FSHD2 pathology occurs when mutations in *SMCHD1*, *DNMT3B* and/or *LRIF1* (Box 1) cause similar epigenetic changes *in trans* to a *D4Z4* array on a permissive chromosome (Fig. 1A) (de Greef et al., 2009, 2010; Hamanaka et al., 2020; Larsen et al., 2015; Lemmers et al., 2012; van den Boogaard et al., 2016; van Overveld et al., 2003).

**Box 1. Glossary**

**FSHD-associated genetic elements**

**4qA/4qB:** haplotypes located on chromosome 4q adjacent to the *D4Z4* array; 4qA haplotypes are permissive for the disease whereas 4qB haplotypes are not.

***D4Z4* array:** a tandem repeat array composed of 3.3 kb elements; the *D4Z4* array located on chromosome 4q is associated with FSHD.

***D4Z4* unit/repeat:** an individual 3.3 kb element of the *D4Z4* array. Each unit contains one copy of the *DUX4* gene.

**Permissive allele/haplotype/chromosome:** a particular allele or haplotype located on the same chromosome as the *D4Z4* array, but outside the array itself, that is necessary for FSHD to develop (see 4qA/4qB).

**Simple sequence length polymorphisms (SSLPs):** variations in the length of a repeated element. Certain SSLPs are permissive for FSHD.

**Relevant genes and proteins**

**CDK4:** cyclin-dependent kinase 4; used to prevent growth arrest of immortalized myogenic cell lines.

**DUX4:** the gene coding for DUX4 is contained within each *D4Z4* repeat unit. The DUX4 protein is a double homeodomain transcription factor and its expression in muscle is the consensus cause of FSHD.

**DUX4 centromeric (*DUX4c*):** a gene located centromeric to the *D4Z4* array. DUX4c is nearly identical to DUX4 over the N-terminal half of the protein, but has a divergent C-terminus. DUX4c is non-toxic, but has been proposed to contribute to pathology by other mechanisms.

***FRG1*, *FRG2*, *ANT1* and *FAT1*:** genes located near the *D4Z4* array that have been proposed to contribute to pathology.

**Lamin A/C:** protein component of the nuclear matrix. Human-specific anti-lamin A/C antibodies are often used to visualize the human cells in a human-to-mouse xenograft.

**MyHC:** myosin heavy chain; a marker for differentiated myogenic cells.

**PAX3, PAX7, MYOD, MYF5, *myoD*, *myf5* and *pax3*:** master regulatory transcription factors that regulate muscle development.

**SMCHD1, DNMT3B and LRIF1:** proteins that act *in* *trans* to regulate the epigenetic state of the *D4Z4* array. Mutations in the genes coding for these proteins are associated with FSHD2 and can be modifiers of severity in FSHD1.

**Spectrin:** a protein found in mature myofibers. Staining for the human-specific version of this protein can distinguish human from murine fibers in xenografts.

***TERT*:** the gene coding for human telomerase; used to immortalize cell lines.

**Muscle lineage cell types**

**C2C12:** a commonly used murine myogenic cell line.

**Myoblasts:** proliferative mononuclear muscle-lineage cells that arise from satellite cells and are capable of fusing and differentiating into mature muscle fibers.

**Myocytes:** mature, contractile muscle cells that arise from the further differentiation of myotubes.

**Myotubes:** multinucleated post-mitotic cells that arise from the fusion and differentiation of myoblasts.

**Rhabdomyosarcoma cell line:** a cell line isolated from a type of cancer that develops from myogenic lineage cells.

**Satellite cells:** stem cells present in a quiescent state in mature muscle that are capable of differentiating along the myogenic lineage and regenerating muscle tissue following an injury.

**Miscellaneous**

**Adeno-associated virus (AAV):** a viral vector used to deliver genetic constructs to live animals. The number (i.e. AAV6) indicates the serotype of the capsid, which affects its ability to infect different tissues.

**CD56:** a cell surface protein marker used to isolate myogenic cells.

**Embryonic stem cell (ESC):** a pluripotent stem cell isolated from an embryo that can differentiate into many different cell types.

**Homeodomain:** one type of protein motif capable of binding to specific DNA sequences. DUX4 contains two homeodomains, often referred to as HOX domains.

**Human pluripotent stem cell (hPSC):** a stem cell of human origin that can differentiate into many different cell types.

**Induced pluripotent stem cell (iPSC):** a pluripotent stem cell that was generated by reprogramming a differentiated cell.

**Lymphoblastoid cell line (LCL):** an immortalized cell line derived from lymphocytes.

**Myonucleus:** a single nucleus within a multinucleated muscle

Each *D4Z4* unit contains a copy of the *DUX4* (Box 1) gene, which encodes a double homeobox transcriptional activator (Dixit et al., 2007; Gabriëls et al., 1999; Hewitt et al., 1994; Kowaljow et al., 2007; Snider et al., 2009). De-repression of the array alone is not pathogenic, as the *DUX4* transcript does not include a polyadenylation site and will be degraded (Fig. 1A). However, in the presence of a permissive allele, transcription of *DUX4* from the final repeat can read through into the adjacent region and incorporate a suboptimal polyadenylation signal, thereby stabilizing the transcript and allowing it to be processed into mature full-length mRNA (*DUX4-fl*), which is associated with pathology, and into a shorter mRNA (*DUX4-s*), which is not (Dixit et al., 2007; Lemmers et al., 2010b; Snider et al., 2009, 2010) (Fig. 1B). Even in these circumstances, expression of *DUX4* is both rare and sporadic, with DUX4 found in as few as 1 in 2000 FSHD myoblasts and 1 in 200 myonuclei in FSHD myotubes (Box 1) (Block et al., 2013; Jones et al., 2012; Rickard et al., 2015; Tassin et al., 2013; van den Heuvel et al., 2018).

How DUX4 expression leads to pathology in muscle is not completely clear, but the most generally accepted model is that DUX4 activates an improper and pathogenic genetic program in myogenic cells (DeSimone et al., 2017). *DUX4* is normally expressed in the germline, pre-implantation embryo and mesenchymal stromal cells, and its biological function appears to be in regulating development of the very-early-stage embryo and in osteogenic differentiation (De Iaco et al., 2017; de la Kethulle de Ryhove et al., 2015; Hendrickson et al., 2017; Snider et al., 2010). DUX4-target genes include factors associated with zygotic genome activation, cleavage-specific genes, stem cells and germline genes, but, more curiously, they also include immune modulators and non-coding transcripts, including retrotransposons and repetitive elements (De Iaco et al., 2017; Geng et al., 2012; Hendrickson et al., 2017; Rickard et al., 2015; Shadle et al., 2019; Sharma et al., 2013; van den Heuvel et al., 2018; Wong et al., 2020; Young et al., 2013; Zhang et al., 2016). It is likely that these embryonic and non-coding transcripts in the context of muscle cause progressive death of myofibers, as *DUX4* expression is toxic in many models of FSHD, including human and murine cell cultures and several animal models (Block et al., 2013; Bosnakovski et al., 2008b; Dandapat et al., 2014; Jagannathan et al., 2016; Jones et al., 2016; Kowaljow et al., 2007; Mitsuhashi et al., 2013; Rickard et al., 2015; Wallace et al., 2011; Wuebbles et al., 2010). Cell death appears to occur via p53 (also known as TP53) and caspase 3/7 activation (Bosnakovski et al., 2008b; DeSimone et al., 2019; Kowaljow et al., 2007; Lek et al., 2020; Wallace et al., 2011), although p53-independent mechanisms have been proposed (Bosnakovski et al., 2017b; Shadle et al., 2017). Exactly how DUX4 triggers cell death has been a subject of much investigation, and evidence exists for the involvement of many pathways, including oxidative stress (Barro et al., 2010; Bosnakovski et al., 2008b; Bou Saada et al., 2016; Cheli et al., 2011; Dmitriev et al., 2016; Sharma et al., 2013; Turki et al., 2012; Winokur et al., 2003a), mRNA processing and quality control (Feng et al., 2015; Rickard et al., 2015), impairment of the ubiquitin/proteasome pathway (Homma et al., 2015), aggregation of the nuclear proteins TDP-43 and FUS and disruption of nuclear PML bodies and SC35 speckles (Homma et al., 2015, 2016), accumulation of toxic double-stranded RNAs (Shadle et al., 2017; Shadle et al., 2019), hyaluronic acid signaling (DeSimone et al., 2019) and hypoxia/HIF1α pathways (Lek et al., 2020). DUX4 is also associated with a number of other cellular phenotypes that may contribute to pathology, such as myoblast differentiation/fusion defects and altered morphology (Banerji et al., 2018; Barro et al., 2010; Bosnakovski et al., 2008b, 2017c, 2018; Dandapat et al., 2014; Knopp et al., 2016; Tassin et al., 2012; Vanderplanck et al., 2011; Winokur et al., 2003b; Yip and Picketts, 2003), altered β-catenin signaling (Banerji et al., 2015), changes to proteomes (Celegato et al., 2006; Jagannathan et al., 2019; Tassin et al., 2012) and an altered myogenic program (Bosnakovski et al., 2008b, 2017c; Bosnakovski et al., 2018; Celegato et al., 2006; Knopp et al., 2016; Winokur et al., 2003b; Wuebbles et al., 2010). Specifically, DUX4 seems to compete with or suppress the expression of PAX3 and PAX7 (Box 1), and loss of PAX7-target gene expression is a signature of FSHD muscle (Banerji and Zammit, 2019; Banerji et al., 2017; Bosnakovski et al., 2008b, 2017c; Haynes et al., 2017). Also, FSHD muscle biopsies show evidence of immune infiltration (Arahata et al., 1995; Frisullo et al., 2011; Hauerslev et al., 2013; Statland et al., 2015). DUX4 has been detected in lymphoblastoid cell lines (LCLs; Box 1) (Banerji et al., 2020; Jones et al., 2017) and activates immune markers (Geng et al., 2012; Shadle et al., 2017; Wong et al., 2020), which suggests that FSHD might involve an immune component.

In addition to *DUX4*, other genes located near the *D4Z4* array have been proposed to play a role in FSHD, although their pathogenic impact remains controversial (reviewed in DeSimone et al., 2017). In particular, FSHD region gene 1 (*FRG1*), but not the nearby *FRG2* or *ANT1* (also known as *SLC25A4*) (Box 1), showed FSHD-like pathology and impaired muscle growth when overexpressed in mouse (Feeney et al., 2015; Gabellini et al., 2006; Xynos et al., 2013). Similar observations were made in *Drosophila* (Jones et al., 2016), while overexpression of its homolog in *Xenopus* caused vascular abnormalities (Wuebbles et al., 2009). Additionally, *FRG1* overexpression can cause defects in satellite cell (Box 1) function (Xynos et al., 2013) and impaired myogenesis and proliferation in myoblasts (Chen et al., 2011; Feeney et al., 2015; Neguembor et al., 2013). *FAT1* (Box 1) has been proposed to be a modifier of pathology, as muscle-specific loss of *FAT1* in mouse results in phenotypes reminiscent of FSHD (Caruso et al., 2013). *FAT1* variants have been associated with FSHD-like pathology (Park et al., 2018; Puppo et al., 2015), and low *FAT1* expression correlates with earlier-affected muscles in FSHD (Mariot et al., 2015). Another gene in the region, *DUX4c* (Box 1), has an identical N-terminus and homeodomains (Box 1) to *DUX4*, but a divergent C-terminus, and is upregulated in FSHD myoblasts and biopsies (Ansseau et al., 2006, 2009). Unlike DUX4, DUX4c does not appear to be toxic to C2C12 cells (Box 1) (Bosnakovski et al., 2008b) or to *Xenopus* (Wuebbles et al., 2010). However, DUX4c has been associated with changes in myogenic fusion and differentiation, proliferation and misregulation of myogenic factors including MYOD (also known as MYOD1), PAX7 and MYF5 (Box 1) (Ansseau et al., 2009; Bosnakovski et al., 2008a; Knopp et al., 2016). DUX4c also activates expression of FSHD-associated microRNAs (Dmitriev et al., 2013).

There is currently no effective treatment for FSHD, which means that continued studies are critical to the search for therapeutics. The field acutely needs meaningful systems to model the various aspects of the disease and to provide a platform for the testing of new therapeutics. Researchers have developed many cellular and animal models of FSHD, each modeling different aspects of pathology (reviewed in DeSimone et al., 2017; Lek et al., 2015). However, the genetic and epigenetic complexity of the disease make it challenging to model. In particular, the sporadic expression pattern of *DUX4* is very difficult to replicate. Additionally, the toxicity of DUX4 makes modeling FSHD even more challenging, both *in vitro* and *in vivo*, as its expression is quickly followed by cell death, meaning that models are difficult to propagate and offer only a small window to study DUX4 activity. Fortunately, recent development of new models and continued iterations of existing ones has led to several new systems that are much more relevant than was achieved previously. Here, we review the development of *DUX4-*based models of FSHD, their advantages and how they can accelerate the discovery of treatments for FSHD. Alternative models of FSHD that are not based on *DUX4* have also been developed, but will not be discussed here, as they have been reviewed previously (DeSimone et al., 2017; Lek et al., 2015).

## Cellular models of FSHD

**Fig. 2.**
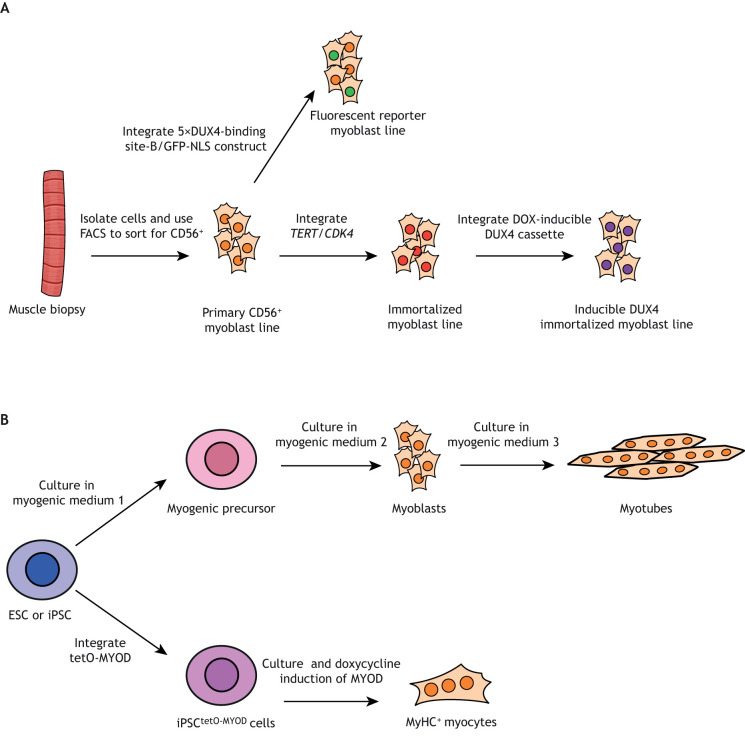
**Cellular models of FSHD.** (A) Cells are isolated and cultured from donated biopsies from FSHD patients or control subjects. Cultures are enriched for myogenic cells using fluorescence-activated cell sorting (FACS) (Homma et al., 2012; Jones et al., 2012). These primary cell populations, composed chiefly of proliferating myoblasts, can be propagated for experiments and/or deposited in a cell repository. Primary CD56^+^ (Box 1) cell lines can be modified by the integration of a DUX4 fluorescent reporter construct (Rickard et al., 2015), or they can be immortalized by integrating *TERT* and *CDK4* expression cassettes (Krom et al., 2012; Stadler et al., 2011, 2013). Immortalized cell lines can be further modified by integrating inducible *DUX4* constructs. (B) Embryonic stem cells (ESCs) or induced pluripotent stem cells (iPSCs) donated by FSHD patients can be used to generate myogenic lineage cells by culturing in three specific media to differentiate into myogenic precursors, then myoblasts, then myotubes (Caron et al., 2016). Alternatively, an inducible MYOD construct can be integrated into iPSCs, which prompts the cells to differentiate into MyHC^+^ myocytes by culturing in doxycycline-containing medium (Sasaki-Honda et al., 2018). tetO, tetracycline operator.

*In vitro* studies are essential for investigating the molecular and biochemical underpinnings of disease. Although researchers frequently use well-established cell lines such as HeLa and HEK293, modeling disease in the most meaningful way requires experiments in the relevant cell type. Because mature muscle is post-mitotic, researchers typically use myoblast cell lines, as they can be propagated and induced to fuse into myotubes as needed. However, there are additional challenges particular to FSHD. As discussed above, *DUX4* is expressed in a very small proportion of cells in FSHD, and its toxicity results in transient expression of the protein, making it difficult to detect in cell culture. Furthermore, because of the genetic heterogeneity of FSHD, observations in cells derived from one FSHD patient may not be representative of the larger FSHD population. Researchers have developed several approaches to design cell culture systems to address these issues, and although no one system overcomes them all, these have contributed greatly to accelerating FSHD research. These cellular models of FSHD are discussed in this section and summarized in Fig. 2.

### Cell repositories

Perhaps the most straightforward way to address genetic heterogeneity in FSHD is a large bank of FSHD patient-derived cell lines from diverse genetic backgrounds, so that studies can be performed on materials from different patients simultaneously. Such a resource was created by deriving primary myogenic cell lines from a large group of FSHD patients (Homma et al., 2012; Jones et al., 2012). Biceps and deltoid biopsies were taken from patients and their first-degree unaffected relatives, and their *D4Z4* repeat size, age at clinical onset and at biopsy, and biceps and deltoid strength were recorded. Primary myogenic cells were derived from these biopsies by enriching for CD56-positive cells (Fig. 2; Box 1), and are available to researchers via a repository at the Wellstone Center for FSHD Research at the University of Massachusetts Medical School, Worcester, MA, USA. Additional repositories of biological materials include the Richard Fields Center for FSHD Research at the University of Rochester Medical Center, Rochester, NY, USA, and the Wellstone Muscular Dystrophy Specialized Research Center at the University of Iowa, Iowa City, IA, USA.

Although primary cell lines are powerful tools for studying FSHD, they are, unfortunately, a limited resource, as they undergo replicative senescence and need to be re-isolated from fresh biopsies. To overcome this, Stadler and colleagues have developed a procedure to immortalize these lines. In this system, myoblasts are transduced with *TERT* and *CDK4* (Box 1), which prevent telomere shortening and growth arrest, to create immortalized versions of cell lines from previous studies (Stadler et al., 2011, 2013) and isogenic lines derived from mosaic FSHD patients bearing contracted and uncontracted *D4Z4* arrays (Krom et al., 2012). This procedure expands the usefulness of cell lines, although some lines did show diminished differentiation ability after very long periods of culturing. These immortalized cell lines are also included in cell repositories.

### LCLs

In addition to FSHD patient-derived myogenic cell lines, a collection of 114 immortalized FSHD-affected and healthy control subject-derived LCLs from 12 multigenerational FSHD families has recently been established, and is deposited in the NIGMS Human Genetic Cell Repository at the Coriell Institute for Medical Research, Camden, NJ, USA (Jones et al., 2017). Critical information including clinical FSHD status, *D4Z4* repeat length and epigenetic status, 4qA/4qB (Box 1) allele status and *DUX4-fl* expression have been determined for each line, and family pedigrees are available, making this a particularly informative collection of biological materials. Even though LCLs are not myogenic in nature, only FSHD patient-derived lines express *DUX4* (Jones et al., 2017), and the DUX4 transcriptome in LCLs is similar to that in myogenic lineages (Banerji et al., 2020), suggesting that LCLs are appropriate for studying DUX4 expression and activity. Importantly, the specific expression of *DUX4-fl* in LCLs supports the hypothesis that FSHD pathology involves an immune component, and these LCLs provide a model for studying the effects of DUX4 in immune-lineage cell lines.

### Fluorescent reporter cell lines

To address the technical challenges of the infrequent *DUX4* expression in patient-derived cell lines, Rickard et al. (2015) developed a reporter system that allows easy identification of endogenous *DUX4*-expressing cells. The system uses a lentivirus to integrate a construct containing five copies of the DUX4-binding site driving expression of a nuclear localization signal-tagged green (GFP) or blue (BFP) fluorescent protein. This fluorescently labels the rare *DUX4*-expressing patient-derived myoblasts or myonuclei so they can easily be identified, thereby enabling studies specifically on the *DUX4*-expressing subpopulation. This powerful system can be integrated into any cell line and was the first to demonstrate that endogenous levels of DUX4 are myotoxic and to establish the DUX4 transcriptome in its native environment (Rickard et al., 2015).

### Human pluripotent stem cell- and induced pluripotent stem cell-derived myogenic cell lines

In an alternative approach to address the limited lifespan of primary cell lines, Caron et al. (2016) developed a human pluripotent stem cell (hPSC; Box 1)-based FSHD model. FSHD-affected or unaffected embryonic stem cells (ESCs; Box 1) were induced to differentiate into myogenic cells through a three-step culturing protocol. Cells are cultured in three defined media that induce ESC differentiation along the myogenic lineage, first producing myogenic precursors, then myoblasts, then myotubes (Caron et al., 2016). Notably, this protocol can be completed in a shorter timeframe than other myogenic differentiation protocols and involves no genetic manipulations. Cell lines can be isolated at any step, allowing researchers to establish myoblast lines. The cells differentiated using this protocol have very similar gene expression profiles and functional properties to muscle biopsy-derived lines. Critically, this protocol can also be used on induced pluripotent stem cells (iPSCs; Box 1), creating the potential for deriving myogenic cell lines from nearly any cell type, thereby reducing the need for muscle biopsies.

In an alternative model system, Sasaki-Honda et al. (2018) isolated non-myogenic cells from an FSHD1 and an FSHD2 patient, and an unaffected control donor, reprogrammed them to iPSCs, and then transfected them with a vector carrying a tetracycline-inducible MYOD. The iPSCs could then be differentiated into MyHC-positive myocytes (Box 1) by inducing MYOD expression with doxycycline (DOX). The resulting FSHD patient-derived myocytes, but not the control-derived ones, expressed *DUX4* as well as several of its target genes, and were used to model the effects of oxidative stress on *DUX4* expression. Thus, this system provides a convenient way of generating cultured myocytes without muscle biopsies and avoids the replicative senescence associated with long-term culturing of cell lines.

### DUX4-inducible cell lines

Several of the approaches described above provide ways to generate large volumes of genetically diverse FSHD patient-derived cell lines, but they do not address the low frequency of DUX4 expression. To overcome this issue, researchers have generated myoblast lines that carry inducible *DUX4* transgenes that allow the lines to be propagated without expressing DUX4, but allow robust and uniform expression of *DUX4* on demand. The first of these was produced by Bosnakovski et al. (2008b), who integrated a DOX-inducible *DUX4* transgene into C2C12 cells. The resulting iC2C12-DUX4 line allowed rapid and titratable expression of DUX4 through the addition of DOX to the culture medium, and immediately proved useful for identifying DUX4-target genes and for studying the effects of DUX4 expression in myogenic cells, including myogenesis, oxidative stress and toxicity.

Although the iC2C12-DUX4 system is quite powerful, interpretation of results is limited because a murine cell line is likely not representative of human myoblasts. This issue has been overcome by the development of two immortalized human myoblast cell lines carrying DOX-inducible *DUX4* transgenes. Choi et al. (2016) integrated a DOX-inducible, FLAG-tagged *DUX4* into the LHCN-M2 myoblast line. The resulting LHCN-M2-iDUX4 cells showed similar rapid activation of DUX4 expression and similar phenotypes as the iC2C12-DUX4 line. Similarly, the LHCN-M2-iDUX4 line was also titratable and suitable for studying DUX4 under low-expression conditions (Bosnakovski et al., 2018). In an alternative approach, Jagannathan and Shadle et al. developed a DOX-inducible model by altering the coding sequence of DUX4 to remove as many CpGs as possible without altering the protein sequence, which prevented epigenetic silencing of the construct, and integrated it into immortalized MB135 human myoblasts (Jagannathan et al., 2016). The resulting MB135-DUX4i cell line was extensively characterized, and found to closely replicate the gene expression profile of other DUX4 expression systems, including endogenously DUX4-expressing fusion-blocked myotubes, making it an excellent cell model for FSHD that has been widely adopted by the research community. Additionally, DOX-inducible DUX4 has also been established in a rhabdomyosarcoma cell line (Box 1) that was used to study DUX4 in a p53-null background and to demonstrate the existence of a DUX4-dependent, p53-independent, toxicity pathway (Shadle et al., 2017).

## Animal models of FSHD

Animal models of disease are a critical tool for the development of therapeutics, as they provide systems that recapitulate diseases at molecular, physiological and functional levels. Developing an animal model for a disease such as FSHD, which arises from inappropriate expression of an endogenous gene, requires stable and controllable expression of the disease-causing gene in a system that can be propagated. The most straightforward way would be to design a conditional expression system of the orthologous disease gene in a chosen animal. Unfortunately, the *D4Z4* array and *DUX4* are only strongly conserved in primates (Clapp et al., 2007; Leidenroth et al., 2012), and the relevance of *DUX4* paralogs to FSHD is uncertain. For example, overexpression of the murine *Duxbl* (also known as *Duxbl1*) is not myotoxic (Eidahl et al., 2016). Furthermore, while overexpression of the murine *Dux* does cause some toxicity, it only shares partial sequence homology to *DUX4*, mostly in its homeodomains, a partial set of common binding sites and target genes, and activates a divergent set of retrotransposons (Eidahl et al., 2016; Whiddon et al., 2017). Nevertheless, the critical need for FSHD models has motivated several attempts to generate *in vivo* model systems, typically by expressing human *DUX4* in the muscle of the animal. These approaches have gone through several iterations, and there are now multiple systems that successfully model several aspects of the disease, which we discuss below and are summarized in Table 1.

**Table 1.**
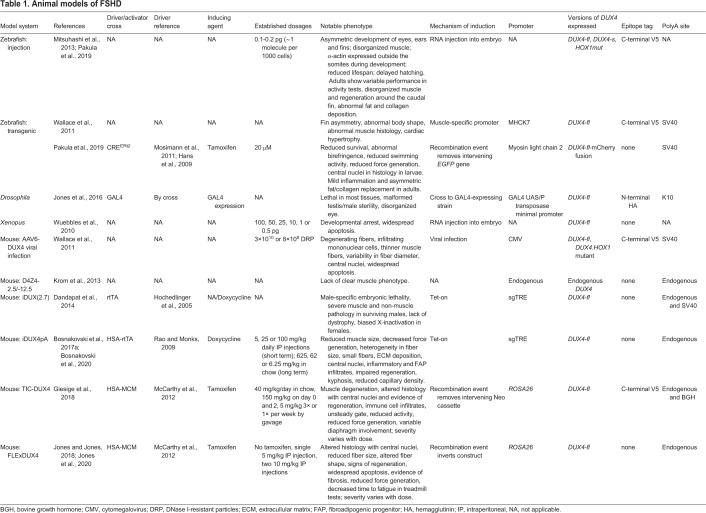
**Animal models of FSHD**

### Zebrafish injection model

Zebrafish (*Danio rerio*) injection models for FSHD involve injecting mRNA into one- or two-cell stage embryos and then observing developmental or muscle abnormalities (Mitsuhashi et al., 2013; Snider et al., 2009). The best-characterized of these was developed by Mitsuhashi et al. (2013), in which they injected one-cell stage embryos with either full-length *DUX4* mRNA (*DUX4-fl*), short *DUX4* mRNA (*DUX4-s*) or *DUX4* mRNA with a defective DNA-binding domain (*HOX1mut*). *DUX4-fl*, but not the others, was highly toxic to the embryos, confirming the toxicity of the full-length DUX4 protein. To better replicate the mosaic pattern of DUX4 expression in FSHD patients, the authors titrated RNA to achieve a ratio of ∼1 mRNA molecule per 1000 cells at the shield/bud stage of development. At these levels, *DUX4-fl* mRNA was still toxic, although to a lesser degree, and caused asymmetric abnormalities in the eyes, ears and fins of the zebrafish larvae. Additionally, birefringence and immunostaining assays revealed disorganized muscle in the *DUX4-fl*-injected zebrafish and, using a previously described EGFP reporter (Hsiao et al., 2001), demonstrated that *DUX4-fl* caused asymmetric defects in muscle development, including ectopic expression outside the somites (Mitsuhashi et al., 2013).

The Mitsuhashi et al. model is a valuable tool for studying FSHD. Although this mRNA-based system cannot be used to study disease genetics, it recapitulates the sporadic expression pattern of *DUX4* quite well and reproduces the asymmetric phenotypes associated with FSHD. Thus, it provides an excellent model to test therapeutic compounds (Lek et al., 2020). Furthermore, this system can be used to investigate developmental aspects of the disease, as it provides a single pulse of *DUX4* expression early in development, which can cause muscle disorganization and other phenotypes in adult zebrafish (Pakula et al., 2019).

### Transgenic zebrafish models

An alternative approach to introduce the human *DUX4* gene to zebrafish is to generate transgenic animals. Wallace et al. (2011) developed the first such model by integrating a cassette containing a V5-tagged *DUX4* under the control of a muscle-specific promoter. The *DUX4*-expressing embryos, but not GFP-expressing controls, displayed severe phenotypes, including fin asymmetry, abnormal body shape and abnormal muscle histology. Curiously, transgenic fish also showed cardiac hypertrophy, which is not present in FSHD.

A second transgenic zebrafish model developed by Pakula et al. (2019) provides more control over the expression of its *DUX4* transgene. This system uses a myosin light chain 2 promoter driving the expression of *EGFP* flanked by two loxP sites, followed by an mCherry-tagged *DUX4*. When crossed with a line expressing a tamoxifen-inducible Cre recombinase (Hans et al., 2009; Mosimann et al., 2011), *EGFP* is excised and recombination enables the expression of the DUX4-mCherry fusion (Pakula et al., 2019). This strategy enables control over the timing of DUX4 expression and monitoring of its spatiotemporal properties via fluorescent reporters. Tamoxifen titration achieves a mosaic pattern of DUX4 expression to recapitulate patient muscle tissue. In this model, DUX4-expressing larvae showed muscle phenotypes including abnormalities in birefringence, altered swimming behavior, histology, central nuclei, and lowered twitch and tetanic force, while adults showed small changes to muscle ultrastructure, mild inflammation, asymmetric replacement of muscle fibers with fat and collagen, and variable changes in swimming speed. This model is useful for studying FSHD progression. It has the advantages of re-creating the mosaic pattern of DUX4 expression, easily assessable muscle phenotypes, variable DUX4 dosages, and the potential for assessing the effects of DUX4 or candidate FSHD treatments over the animal's lifespan.

Both the mRNA injection and transgenic zebrafish models have been used for therapeutic testing of candidate small molecule compounds (Lek et al., 2020). However, given that both models lack the endogenous regulatory regions and genomic context of *DUX4*, these models are not ideal for studies of FSHD genetics and/or epigenetics, and cannot be used to evaluate antisense oligonucleotide-based therapeutics that target the untranslated regions of the *DUX4* transcript.

### *Xenopus* injection model

One of the earliest attempts to model FSHD was the *Xenopus* injection model developed by Wuebbles and Long et al. The authors injected *DUX4* mRNA into one side of four-cell stage *Xenopus* embryos, while the other side was left uninjected (Wuebbles et al., 2010). DUX4 was highly toxic, causing widespread apoptosis and developmental arrest, even at low doses. Developing embryos showed loss of muscle, and *myoD*, *myf5* and *pax3* (Box 1) were missing from most of the injected embryos. The lack of a robust muscle-specific phenotype and developmental arrest has limited the utility of this system for further FSHD modeling.

### Transgenic *Drosophila* model

Jones et al. (2016) developed a transgenic fruit fly to model FSHD by placing a hemagglutinin (HA)-tagged, *Drosophila* codon-optimized full-length *DUX4* under the control of the GAL4 upstream activating sequence (UAS). This system allows *DUX4* to remain silent until the fly line is crossed with a source of GAL4. Many tissue-specific GAL4 fly strains are available, giving this system great flexibility in controlling the timing and location of *DUX4* expression. Unfortunately, the flies displayed phenotypes that limit its usefulness as an FSHD model. Both ubiquitous and adult muscle-specific expression of *DUX4* were lethal. Expressing *DUX4* in the germline resulted in sterility and malformed testes in the males, whereas the females showed no effect. This is a troublesome phenotype for FSHD modeling, as *DUX4* is normally expressed in human testes (Snider et al., 2010). Restricting expression to the eye resulted in reduced lethality, loss of corneal lenses and pigment cells, and disorganized ommatidia and bristles, which the authors propose could be used to screen for enhancers or suppressors of DUX4 function (Jones et al., 2016).

### AAV6-DUX4 mice

Among the first attempts to develop a murine *in vivo* model for FSHD was the adeno-associated virus (AAV; Box 1)-based approach developed by Wallace et al. (2011). This system consisted of a cytomegalovirus (CMV) promoter-driven V5-tagged DUX4 followed by an SV40 polyadenylation site contained within an AAV vector. Injecting the virus into the tibialis anterior of a mouse resulted in DUX4 expression and signs of muscle degeneration, including degenerating myofibers, infiltration of mononuclear cells and p53-depenent apoptosis, as well as evidence of muscle regeneration. The strength of this system is in overcoming the low frequency of *DUX4* expression, thereby allowing observation of the effects of DUX4, and the degree of expression can be adjusted by titrating the virus. Additionally, this system provides the opportunity to study DUX4 in greater molecular detail, as mutations can be readily introduced into the viral construct. For example, point mutations in the DUX4 HOX domain reduce its ability to cause extensive muscle damage and apoptosis (Wallace et al., 2011). Also, this system allows co-infection experiments, which have shown that microRNAs can protect muscle from DUX4-induced pathologies (Wallace et al., 2012, 2018).

Although this viral infection model has been used to great effect, it has limitations. DUX4 expression remains localized to the specific muscle injected, and thus many global measures of muscle function cannot be assessed. Additionally, because DUX4 is toxic, the transduced fibers will die and will be replaced by newly generated untransduced fibers, and mice can partially recover by 3 weeks post-injection (Wallace et al., 2011), making this model inappropriate for longer-term experiments.

### D4Z4-2.5 and D4Z4-12.5 mice

To generate a transgenic mouse model of FSHD, Krom et al. (2013) developed a pair of transgenic mice that contain fragments of the human D4Z4 region. The D4Z4-2.5 mouse contains four copies of an FSHD patient-derived sequence, each containing 2.5 repeats of the *D4Z4* array, as well as the endogenous *DUX4* polyadenylation signal on a permissive 4qA haplotype. The D4Z4-12.5 mouse contains an integrated construct containing 12.5 *D4Z4* array repeats on a permissive 4qA allele, which is non-pathogenic, as well as single copies of nearby *FRG1* and *FRG2* genes.

The D4Z4-2.5 mouse displays a pattern of *DUX4* expression that reflects those observed in patients, with variable levels of *DUX4* mRNA in the testes, embryonic stem cells and a panel of skeletal muscle tissues. In D4Z4-12.5 mice, *DUX4* was consistently found only in the testes and, at much lower levels, in some muscle tissues. These mice also recapitulated much of the epigenetics of the disease, with the array in D4Z4-2.5 mice showing relatively relaxed chromatin and hypomethylation compared to D4Z4-12.5 mice. Unfortunately, this system was less successful at modeling disease physiology, as muscles appeared histologically and functionally normal. Therefore, these mice are useful for modeling specific molecular aspects of FSHD, such as *DUX4* expression during muscle regeneration (Knopp et al., 2016) or effects of *SMCHD1* mutations on the *D4Z4* array (de Greef et al., 2017).

### iDUX(2.7) and iDUX4pA mice

Dandapat and Bosnakovski et al. developed a transgenic mouse that carried a DOX-inducible *DUX4* and downstream sequences followed by an SV40 polyadenylation signal on the X chromosome (Dandapat et al., 2014). Unfortunately, this system did not provide the intended characteristics. Very few males survived to birth, and those that did displayed a number of phenotypes including runting, flaky skin, alopecia, high-frequency hearing loss, changes in respiration and activity, and a reduced lifespan (Dandapat et al., 2014, 2016). Although the muscles of these mice showed many signs of pathology, they were not dystrophic, limiting their usefulness as an FSHD model. Females showed only mild phenotypes, apparently due to selective inactivation of X chromosomes carrying the iDUX4(2.7) construct (Dandapat et al., 2014). The phenotypes in these mice were attributed to leaky expression of the transgene in uninduced animals.

To overcome the shortcomings of the iDUX(2.7) mouse, the same group developed the iDUX4pA model (Bosnakovski et al., 2017a). In this iteration, the authors removed the SV40 polyadenylation signal, leaving only the less efficient endogenous site in the DUX4 3′ untranslated region. This markedly changed the phenotypes of male iDUX4pA mice. Male carriers were born at near-normal Mendelian ratios, had only a modestly reduced body weight and lived up to 4 months, although the skin and hearing loss phenotypes remained. These mice had milder muscle phenotypes and did not show signs of dystrophy.

Unfortunately, inducing *DUX4* expression with DOX resulted in death of the animals within 24 h, which necessitated a second change to the model. The ubiquitously expressed Tet-on system (Hochedlinger et al., 2005) was replaced with a muscle-specific one (Rao and Monks, 2009). This change allowed more accurate modeling of FSHD pathology (Bosnakovski et al., 2017a). DUX4 induction caused muscle mass loss and significant muscle force decline. Importantly, signs of dystrophy appeared, including many small, damaged and necrotic fibers, central nuclei, the beginnings of fibrosis, immune cell infiltration and impaired regeneration. The system proved useful at the molecular level as well, because *DUX4* expression was sporadic in only a small fraction of fibers and DUX4-target genes were expressed specifically in muscle following DOX induction.

Although the clinical relevance of the uninduced state is unclear, the DOX-induced state reproduces many aspects of the disease, making iDUX4pA a successful FSHD model. DUX4 induction was achieved in both males and females, allowing modeling of potential sex-specific effects of therapeutics. Importantly, many of the phenotypes were dose dependent, which provides the opportunity to titrate for phenotype severity and to adjust the timing of induction. Additionally, this model has proven useful for long-term modeling of FSHD (Bosnakovski et al., 2020). Providing the mice with a low dose of DOX in the chow for up to 6 months produced a progressive muscular dystrophy phenotype that recapitulated many aspects of FSHD, including loss of force generation, histological signs of dystrophy, infiltration of inflammatory and fibroadipogenic progenitor cells, compromised vasculature, fibrosis, and a gene expression signature similar to that found in patients. This therefore enables the system to model chronic aspects of FSHD, or to test the effects of therapeutics administered constantly over a longer period. This model system is very well suited to test the efficacy of FSHD therapeutics at both molecular and physiological levels, and has already been used to study the therapeutic potential of an inhibitor of the epigenetic regulator p300 (also known as EP300) (Bosnakovski et al., 2019).

### TIC-DUX4 mouse

In an alternative attempt, Giesige et al. developed a tamoxifen-inducible DUX4 mouse model. In this system, a V5-tagged *DUX4* open reading frame and its 3′ untranslated region, including its endogenous polyadenylation signal, followed by a bovine growth hormone polyadenylation site, were integrated into the *ROSA26* locus (Giesige et al., 2018). To prevent leaky expression of the construct, a neomycin (Neo) resistance cassette flanked by LoxP sites was placed upstream of the *DUX4* construct, physically separating it from the *ROSA26* promoter. This created a system in which the *DUX4* transgene is not expressed until the introduction of Cre recombinase, which would excise the Neo cassette and allow the *ROSA26* promoter to drive *DUX4* expression. To provide the Cre recombinase, the authors crossed the DUX4 transgenic mouse with various sources of Cre, eventually selecting a previously described skeletal muscle-specific system that produces a modified Cre that remains inactive until exposed to tamoxifen (McCarthy et al., 2012).

The TIC-DUX4 mouse is a relevant model for many aspects of FSHD. Pups were born at the expected Mendelian ratios, and there were initially no signs of leaky expression of the transgene in uninduced mice, although low-level expression did occur in older animals (Giesige et al., 2018), demonstrating that the system tightly, but not perfectly, repressed the transgene. Upon induction, the TIC-DUX4 mice develop an FSHD-like pathology with muscle degeneration and altered histology, including many central nuclei and reduced fiber diameter, immune cell infiltrates, changes in gait and activity, and reduced force generation. Molecular analysis also confirmed appropriate DUX4 expression in induced, but not in uninduced muscle, and it did not appear in the kidney or liver. A representative DUX4-target gene, *Wfdc3*, also showed muscle-specific expression (Giesige et al., 2018).

Similar to the iDUX4pA mouse, the TIC-DUX4 model also showed flexibility. The authors established seven different tamoxifen dosing regimens that induced a range of phenotypic and histopathological severities and allowed the mice to reach the endpoint criteria at different times. As one low-dosage induction regimen resulted in the mice recovering after several months, care must be taken when using this dosing regimen for longitudinal studies. The utility of the TIC-DUX4 mouse has been demonstrated in a preclinical gene therapy study of AAV1.Follistatin, where treated mice showed improved muscle mass and force generation (Giesige et al., 2018).

### FLExDUX4 mouse

Jones and Jones (2018) developed a mouse model similar to the TIC-DUX4 mouse. This system also integrates *DUX4* at the *ROSA26* locus, but with the key difference of the construct including both 5′ and 3′ untranslated regions of *DUX4*, the endogenous polyadenylation site, and lacking an epitope tag. Additionally, the sequence of the *DUX4* transgene contains several silent point mutations designed to prevent the pre-mRNA splicing into the non-pathogenic *DUX4-s* isoform. Thus, this system enables the investigation and targeting of the non-coding portions of the *DUX4* RNA, such as trials of morpholino-based therapeutics. Also, rather than using a spacer to prevent uncontrolled *DUX4* expression, the cassette has been integrated into the locus in reverse orientation and is flanked by unidirectional recombination sites. When exposed to Cre, the recombination flips the transgene, thereby allowing expression of *DUX4* driven by the *ROSA26* promoter. Also, similarly to the researchers who developed the TIC-DUX4 mouse, Jones and Jones (2018) crossed their transgenic mouse with several Cre-carrying lines to optimize the induction, and the same skeletal muscle-specific, tamoxifen-inducible mouse (McCarthy et al., 2012) produced the best FSHD model.

Although the FLExDUX4 system was designed to prevent leaky expression, hemizygous and homozygous mice did show some phenotypes associated with low-level *DUX4* expression, including alopecia, and a slightly smaller body size in older mice, and some transgene transcripts were detected, but this did not affect the fertility or the viability of the mice. Crossing to the Cre-expressing mouse increased leakage of the *DUX4* transgene, which appeared to be a result of a low basal level of recombination (Jones and Jones, 2018; Jones et al., 2020) and led to a very mild but observable muscle-wasting phenotype. Inducing recombination with tamoxifen, however, resulted in a phenotype that much more closely resembled that of FSHD (Jones and Jones, 2018). Muscles displayed several signs of pathology including variable fiber size, immune cell infiltration, necrosis and phagocytosis, and fibrosis. Although *DUX4* remained difficult to detect directly, immunostaining showed that the protein was expressed in a mosaic pattern in myonuclei, and that DUX4-target gene expression was significantly increased. Similar to other inducible mouse models, the FLExDUX4 mouse also has a large degree of versatility for modeling the variable severity of FSHD. *DUX4* expression can be titrated by adjusting tamoxifen dosage, achieving a range of phenotypic severities (Jones et al., 2020). However, as with the TIC-DUX4 model, mice given moderate doses of tamoxifen recovered from DUX4 induction at later time points, with improved treadmill test outcomes, confirming that researchers must take care when designing experiments.

Overall, the FLExDUX4 mouse has proven to be an excellent model system. It is being widely adopted amongst the FSHD community and has proven useful in demonstrating the effectiveness of LNA gapmer antisense oligonucleotides to reduce *DUX4* expression *in vivo* (Lim et al., 2020) and in showing a sarcolemmal repair defect and decreased torque in *DUX4*-expressing muscle (Bittel et al., 2020).

## Mouse xenograft models of FSHD

**Fig. 3.**
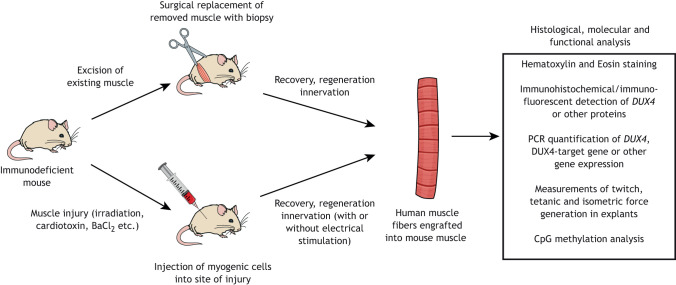
**Xenograft models of FSHD.** Existing muscle tissue is removed from immunodeficient mice, either surgically or by injuring the muscle. A donated human biopsy can then be surgically engrafted into the animal (Zhang et al., 2014), or cultured human myogenic cells can be injected into the injury site (Krom et al., 2012; Moyle et al., 2016; Mueller et al., 2019; Oliva et al., 2019; Sakellariou et al., 2016). Animals are allowed to recover for several weeks while the human myogenic cells expand and generate new human muscle fibers to replace the damaged murine muscle. Efficiency of engraftment may be enhanced using intermittent electrical stimulation (Mueller et al., 2019; Sakellariou et al., 2016), and the established grafts can be used in a number of histological, molecular and functional assays. BaCl_2_, barium chloride.

As we discussed, many current animal models are powerful tools for studying FSHD. Unfortunately, all *DUX4* expression models suffer from the complication that expressing human *DUX4* in a model organism may not truly reflect the condition in human muscle. For example, the degree of overlap between DUX4-target genes in human and mouse has been somewhat controversial, with some studies showing activation of similar target gene sets in mouse and human, while others show more divergent targets (Knopp et al., 2016; Krom et al., 2013; Sharma et al., 2013; Whiddon et al., 2017). For example, DUX4 does not activate MERV-L elements in mouse (Whiddon et al., 2017). To mitigate this, an alternative approach has been to engraft FSHD patient-derived muscle tissue or cultured cells into mice, and to use the grafts to evaluate therapeutics. Although this approach does not have the same drawbacks as transgenic *DUX4*-expressing animals, it is not amenable to many functional tests, and therefore data collection is often limited to histology or molecular outputs. Xenograft and transgenic FSHD models should therefore be thought of as complementary systems. The xenograft models mostly differ in the materials being transplanted, which can be either biopsies or cultured cells, and in the type of injury used to promote engraftment, which can include irradiation, cryoinjury or toxins. The various approaches to modeling FSHD with xenografts are summarized here and in Fig. 3.

### Human biopsy xenografts

The first biopsy xenograft model for FSHD developed by Zhang et al. (2014) involved grafting muscle biopsies from donors into the hindlimb of immunodeficient NOD*-Rag1^null^IL2ry^null^* mice. The xenografts became vascularized and innervated, and they survived up to 41 weeks post-implantation. Interestingly, the original myofibers of the donated biopsies degenerated, but new fibers regenerated within the graft. Extensive immunohistochemistry analyses revealed that the regenerated grafts were composed solely of human myofibers, although the capillaries were of both human and murine origin. Grafts contained both type 1 and type 2 fibers, and although it was not possible to assess the functionality of the graft *in vivo*, explanted xenografts generated twitch, tetanic and isometric force. *DUX4* expression was specifically detected in the grafted muscle, and a panel of DUX4-target genes was upregulated in the FSHD grafts, confirming the suitability of these xenografts as models of FSHD.

Because the biopsy xenograft system relies on endogenous *DUX4* expressed in human muscle fibers, it makes for a highly relevant model, and has been used to test the ability of an antisense phosphorodiamidate morpholino oligonucleotide to repress *DUX4* (Chen et al., 2016). Although powerful, this model does have significant limitations. Most notably, fresh biopsies must be continually donated from FSHD patients. Also, because the xenografts require the use of immunodeficient mice, it is not possible to investigate the contributions of the immune system to pathology.

### Human myogenic cell xenografts

Myogenic cell-based xenograft models have been developed as an alternative to difficult-to-obtain biopsy xenografts, as a single donation can generate large volumes of myogenic cells that can be cultured and implanted as needed. Cell xenograft approaches require that the engrafted cells regenerate, grow and differentiate into new human muscle fibers within an injured murine muscle. This ability was demonstrated in an early xenograft model for FSHD by Krom et al. (2012) that used cryoinjury to damage the tibialis anterior muscle of immunodeficient *Rag2*^−/−^ *γC*^−/−^ (also known as *Il2rg*^−/−^) *C5*^−/−^ (also known as *Hc*^−/−^) mice. Immortalized isogenic myoblasts carrying either contracted or non-contracted *D4Z4* alleles were engrafted into the wound, and, after 1 month, the engrafted muscle was removed, and either cryosectioned or used to isolate RNA. Expression of human-specific lamin A/C and spectrin (Box 1) in tissue sections demonstrated that the engrafted myogenic cell lines carrying contracted alleles retain their ability to incorporate into regenerating muscle fibers, and reverse transcription polymerase chain reaction (RT-PCR) analysis confirmed contraction-specific expression of a DUX4-target gene, thereby showing that myogenic cell xenografts can provide an *in vivo* model for FSHD.

Sakellariou and O'Neill et al. established a xenograft model in which they injected the tibialis anterior of immune-deficient mice with cardiotoxin to eliminate the existing muscle and X-irradiated it to prevent regeneration, and then engrafted immortalized human myogenic precursor cells into the injury (Sakellariou et al., 2016). To promote engraftment and maturation, 5 days after the engraftments, mice underwent intermittent electrical stimulation. After several weeks, innervated muscle fibers of human origin formed with minimal contamination by murine muscle, although they were smaller than mature mouse muscle fibers; the mass of the grafts ranged from 4 mg to 14 mg, compared to mouse tibialis anterior muscles, which ranged from 31 mg to 51 mg. The engrafted myogenic precursor cells derived from FSHD-affected and -unaffected donors formed myofibers of similar size and density (Mueller et al., 2019; Sakellariou et al., 2016). Notably, the grafts contained satellite cells of human origin, suggesting that they could recapitulate the satellite cell niche (Mueller et al., 2019). Expression of *DUX4* and DUX4-target genes were considerably upregulated in FSHD grafts relative to controls, with FSHD grafts showing positive immunostaining for the protein marker SLC34A2, demonstrating that this system recapitulates many established aspects of FSHD (Mueller et al., 2019).

In addition to the Sakellariou et al. system, similar human xenograft systems have proven useful for preclinical testing of FSHD therapeutic compounds. Adapting a previously established barium chloride injury system (Hardy et al., 2016), Oliva and colleagues established a xenograft model for testing FSHD therapeutics (Oliva et al., 2019). In their model, FSHD2 patient-derived myoblasts were engrafted into injured tibialis anterior muscles. *DUX4* expression, assessed by quantitative RT-PCR, peaked 4 days after transplantation, with representative DUX4-target gene(s) peaking after 5-6 days. Using this system, they tested the effects of subcutaneous injections of PH-797804 and of oral administration of losmapimod, two p38 inhibitors. Peak *DUX4* and DUX4-target gene expression was reduced by as much as 90%, overall human cell survival increased approximately fivefold and expression of a myogenic marker was not affected, showing that these drugs can inhibit the expression of *DUX4* in human muscle without affecting differentiation.

Moyle et al. (2016) used a similar model to show that the U.S. Food and Drug Administration (FDA)-approved receptor tyrosine kinase inhibitor Sunitinib improved the regeneration capacity of engrafted FSHD myoblasts. Here, the authors cryoinjured the tibialis anterior and implanted immortalized FSHD patient-derived myoblasts into the injured tissue. After 3 weeks of Sunitinib treatment, the muscle was removed, sectioned, and incubated with human-specific antibodies against lamin A/C and spectrin. Sunitinib-treated mice showed higher levels of both proteins, indicating that treatment with this receptor tyrosine kinase inhibitor increased regenerative capacity, thus confirming the value of xenograft models for development of novel therapeutics and for drug repurposing studies.

## Conclusions

As our understanding of FSHD has progressed, it has intensified the need to establish relevant disease models to enable the translation of new biological insights into therapeutic development. Until recently, FSHD models were largely restricted to a small number of patient-derived cell lines, transgenic models of questionable relevance and non-scalable muscle xenografts. However, work within the past several years has resulted in a variety of patient-derived and engineered *in vitro* and *in vivo* models. These have been rapidly adopted by the FSHD research community and enabled many studies of pathological mechanisms and pre-clinical testing of therapeutics. In particular, these new model systems have provided platforms for studying cutting-edge molecular therapies, including CRISPR- and antisense oligonucleotide-based modulation of *DUX4* or other gene expression (Chen et al., 2016; Giesige et al., 2018; Himeda et al., 2016, 2018; Lim et al., 2015, 2020; Marsollier et al., 2016; Wallace et al., 2012).

Although the current generation of models provides better representations of the disease than were previously available, there is still a need for new, more physiologically relevant models. Many *in vitro* models of FSHD are restricted to myoblasts, rather than the more relevant myotubes, and a three-dimensional *in vitro* FSHD model has yet to be established. Similarly, some aspects of the disease are not well represented in the existing *in vivo* models – none of the transgenic mouse models recapitulate the distinct patchy and asymmetric progression of the disease or the variable age of onset or progression rate. Also, transgenic animals poorly recapitulate the contribution of the human immune system to FSHD, and human-to-mouse xenografts require the use of immunocompromised animals. The adaptation of humanized mouse technology for this disease would represent a major advancement in *in vivo* FSHD modeling.

The complex etiology of the disease and the toxicity of DUX4 have made FSHD a notoriously difficult disease to model, yet recent years have seen a proliferation of new and increasingly physiologically relevant models. This has helped move the FSHD field forward, but the approaches used in these models can also serve as a valuable example of how to design models of other difficult-to-model genetic diseases in the neuromuscular and other fields. The use of promoters that can be regulated with drugs, cell type-specific drivers, and creative recombination-dependent expression systems can be adapted to any disease involving the activation of a toxic or deleterious transcript, and there are likely to be many disease models that take advantage of these tools in the coming years.
